# Complete Genome Sequence of a Novel Recombinant GII.P16-GII.1 Norovirus Associated with a Gastroenteritis Outbreak in Shandong Province, China, in 2017

**DOI:** 10.1128/genomeA.01483-17

**Published:** 2018-02-08

**Authors:** Chunrong Wang, Yuanyun Ao, Jiemei Yu, Xiaolu Xie, Hongyan Deng, Miao Jin, Lanzheng Liu, Zhaojun Duan

**Affiliations:** a Viral Disease Inspection Laboratory, Jinnan Municipal Center for Disease Control and Prevention, Jinan, China; b National Institute for Viral Disease Control and Prevention, Chinese Center for Disease Control and Prevention, Beijing, China; c Peking Union Medical College Hospital, Beijing, China; d Department of Medical Microbiology, Qingdao University Medical College, Qingdao, China

## Abstract

We report here the complete genome sequence of a novel recombinant GII.P16-GII.1 norovirus identified from eight fecal samples collected during an acute gastroenteritis outbreak in Jinan, Shandong Province, China, in 2017. The virus had nucleotide identities of 99% and 91% in the ORF1 and ORF2 genes of related strains, respectively.

## GENOME ANNOUNCEMENT

Noroviruses are a leading cause of acute viral gastroenteritis worldwide (1). Based on viral capsid protein (VP1) sequences, noroviruses are divided into 7 genogroups (GI to GVII), which are subdivided into more than 30 genotypes (2). With the frequent emergence of recombinant noroviruses, the U.S. Centers for Disease Control and Prevention recently developed new genotyping methods for VP1 and RNA-dependent RNA polymerase (RdRp) that increase the ability to conduct worldwide surveillance of noroviruses (3).

Eight fecal samples from patients treated during an acute gastroenteritis outbreak in Jinan, Shandong Province, China, in 2017 were diluted into a 10% (1:10 [wt/vol] ratio) viral suspension with phosphate-buffered saline. Total RNA was extracted from the supernatant using a QIAamp viral RNA minikit (Qiagen). The norovirus was detected by targeting the spanning ORF1 and ORF2 genes as previously described (3). A novel recombinant norovirus was identified and designated GII.P16-GII.1. One strain named SDJN170450 was selected for full-length genome sequencing, as previously reported (3).

The complete genome of SDJN170450 was 7,518 nucleotides (nt), excluding the poly(A) tail. Its genome contained 3 open reading frames (ORFs), as follows: ORF1 encoded a nonstructural polyprotein with a length of 1699 amino acids (aa), and ORF2 and ORF3 encoded VP1 and VP2 with lengths of 535 and 259 aa, respectively. Interestingly, the ORF1 sequence of SDJN170450 had the highest nucleotide identity (99%) with the emerging GII.P16 gene of the GII.P16-GII.2 norovirus that predominated in the winter of 2016 to 2017, while the VP1 sequence had the best BLAST search results of 91% and 98% nt and aa identities, respectively, with those of the GII.1 strain Hu/GII.1/Ascension208/2010/USA (GenBank accession number AFA55174). Therefore, we postulate that this GII.1 virus originated from a recombination event between a newly emerged GII.P16-GII.2 virus and a GII.1 virus. Furthermore, SDJN170450 contained 15 aa mutations in VP1, with 5 aa near histo-blood group antigen binding site II compared to that of the GII.1 prototype strain Hawaii/1971/US (AAB97768). The functional importance of these mutations needs further investigation.

This is the first detection and report of the complete genome sequence of a novel GII.P16-GII.1 norovirus involving a gastroenteritis outbreak. In the last 3 years, rare genotypes have replaced the previously predominant GII.4 genotype (4) as the epidemic viruses causing norovirus outbreaks. These include the novel GII.17 variant in the 2014–2015 season (5) and the GII.P16-GII.2 virus in the 2016–2017 winter season (3), both emergent through the acquisition of a novel RdRp gene (6, 7). Since its first identification in the GII.P16-GII.4_Sydney 2012 norovirus in 2015, the emerging GII.P16 gene has been detected in diverse VP1 genotype viruses (3, 8–10). The GII.P16 protein of the reemerging GII.P16-GII.2 and GII.P16-GII.4_Sydney 2012 viruses contains polymerase mutations near a position known to influence protein function and viral transmission (7, 10). These mutations are also shown in the novel GII.P16-GII.1 norovirus. Therefore, we postulate that the advantageous polymerase and amino acid mutations in the capsid protein have produced a highly transmissible GII.1 virus. Close surveillance of its national and global spread is necessary.

### Accession number(s).

The complete genome sequence of the GII.P16-GII.1 norovirus SDJN170450 has been deposited in GenBank under the accession number MG572182.

## References

[1] Ahmed SM, Hall AJ, Robinson AE, Verhoef L, Premkumar P, Parashar UD, Koopmans M, Lopman BA 2014 Global prevalence of norovirus in cases of gastroenteritis: a systematic review and meta-analysis. Lancet Infect Dis 14:725–730. doi:10.1016/S1473-3099(14)70767-4. 24981041 PMC8006533

[2] Vinjé J 2015 Advances in laboratory methods for detection and typing of norovirus. J Clin Microbiol 53:373–381. doi:10.1128/JCM.01535-14. 24989606 PMC4298492

[3] Ao Y, Wang J, Ling H, He Y, Dong X, Wang X, Peng J, Zhang H, Jin M, Duan Z 2017 Norovirus GII.P16/GII.2-associated gastroenteritis, China, 2016. Emerg Infect Dis 23:1172–1175. doi:10.3201/eid2307.170034. 28430563 PMC5512504

[4] Debbink K, Lindesmith LC, Donaldson EF, Costantini V, Beltramello M, Corti D, Swanstrom J, Lanzavecchia A, Vinjé J, Baric RS 2013 Emergence of new pandemic GII.4 Sydney norovirus strain correlates with escape from herd immunity. J Infect Dis 208:1877–1887. doi:10.1093/infdis/jit370. 23908476 PMC3814837

[5] Jin M, Zhou YK, Xie HP, Fu JG, He YQ, Zhang S, Jing HB, Kong XY, Sun XM, Li HY, Zhang Q, Li K, Zhang YJ, Zhou DQ, Xing WJ, Liao QH, Liu N, Yu HJ, Jiang X, Tan M, Duan ZJ 2016 Characterization of the new GII.17 norovirus variant that emerged recently as the predominant strain in China. J Gen Virol 97:2620–2632. doi:10.1099/jgv.0.000582. 27543110 PMC5756485

[6] Chan MC, Lee N, Hung TN, Kwok K, Cheung K, Tin EK, Lai RW, Nelson EA, Leung TF, Chan PK 2015 Rapid emergence and predominance of a broadly recognizing and fast-evolving norovirus GII.17 variant in late 2014. Nat Commun 6:10061. doi:10.1038/ncomms10061. 26625712 PMC4686777

[7] Tohma K, Lepore CJ, Ford-Siltz LA, Parra GI 2017 Phylogenetic analyses suggest that factors other than the capsid protein play a role in the epidemic potential of GII.2 norovirus. mSphere 2:e00187-17. doi:10.1128/mSphereDirect.00187-17. 28529975 PMC5437133

[8] Matsushima Y, Shimizu T, Ishikawa M, Komane A, Okabe N, Ryo A, Kimura H, Katayama K, Shimizu H 2016 Complete genome sequence of a recombinant GII.P16-GII.4 norovirus detected in Kawasaki City, Japan, in 2016. Genome Announc 4:e01099-16. doi:10.1128/genomeA.01099-16. 27795262 PMC5054331

[9] Cannon JL, Barclay L, Collins NR, Wikswo ME, Castro CJ, Magaña LC, Gregoricus N, Marine RL, Chhabra P, Vinjé J 2017 Genetic and epidemiologic trends of norovirus outbreaks in the United States from 2013 to 2016 demonstrated emergence of novel GII.4 recombinant viruses. J Clin Microbiol 55:2208–2221. doi:10.1128/JCM.00455-17. 28490488 PMC5483924

[10] Ruis C, Roy S, Brown JR, Allen DJ, Goldstein RA, Breuer J 2017 The emerging GII.P16-GII.4 Sydney 2012 norovirus lineage is circulating worldwide, arose by late-2014 and contains polymerase changes that may increase virus transmission. PLoS One 12:e0179572. doi:10.1371/journal.pone.0179572. 28662035 PMC5491022

